# Culturally Attuned Leadership and Employee Behavior During Organizational Change Initiatives in a Developing Economy

**DOI:** 10.3390/bs15030349

**Published:** 2025-03-12

**Authors:** Ibrahim Alusine Kebe, Yingqi Liu, Christian Kahl

**Affiliations:** 1 School of Economics and Management, Beijing Jiaotong University, Beijing 100044, China; liuyq@bjtu.edu.cn (Y.L.); christiankahl@bjtu.edu.cn (C.K.); 2 Institute of Public Administration and Management, University of Sierra Leone, Freetown 999127, Sierra Leone

**Keywords:** transformational leadership, transactional leadership, organizational change, employee performance, culturally adaptive leadership

## Abstract

In an era of rapid market shifts and technological disruption, the success of organizational change rests on the ability of leaders to navigate complex cultural dynamics. This study explores how culturally adaptive leadership can drive employee outcomes in Sierra Leone’s commercial banking sector during periods of change. By integrating transformational and transactional leadership styles with Hofstede’s cultural dimensions theory, which focuses on power distance (respect for authority) and uncertainty avoidance (preference for structure), this research examines how these cultural values influence the relationship between leadership approaches and employee outcomes. Using a cross-sectional design, data were collected from 820 employees across commercial banks in Sierra Leone, with data analyzed using structural equation modeling (SEM). The findings reveal that transformational leadership significantly enhances employee outcomes, specifically in high power distance environments where authority is deeply respected, while transactional leadership proves more effective in high uncertainty avoidance settings, where clear structure and predictability are paramount. The study highlights the complementary nature of these leadership styles, suggesting that effective leaders must adapt their strategies to the cultural context to drive performance. While the cross-sectional design limits causal inference, this research underscores the critical importance of culturally adaptive leadership, recognizing how cultural dimensions shape behavior and promote sustained success during change.

## 1. Introduction

In the contemporary globalized world, organizations in developing economies face unprecedented challenges, such as rapid technological advancements, fierce competition, and the necessity to adjust to ever-changing market demands. This calls for an agile and dynamic approach to organizational transformation, an approach that is closely correlated with how well a leader leads (Kumar Basu, 2015; Schiuma et al., 2024). Effective leadership is essential across all sectors and regions, yet its influence is especially pronounced in developing economies, where distinct socio-cultural elements significantly influence corporate conduct and employee engagement (McNaughton, 2003). Therefore, in emerging economies, where socio-cultural factors deeply influence organizational behavior, leadership effectiveness extends beyond strategic foresight. It must be rooted in a deep understanding of local cultural norms and values to truly resonate with employees and drive meaningful outcomes (Den Hartog & De Hoogh, 2024).

Sierra Leone is a multicultural society in West Africa comprising 18 ethnic groups each with its distinct languages, traditions, and social practices. While sub-cultural diversity exists, shared historical and socio-political structures, such as hierarchical governance systems and post-colonial institutional legacies, promote commonalities in cultural dimensions like power distance and uncertainty avoidance at the national level.

The country is classified as a developing economy due to systemic challenges such as low GDP per capita (USD 461.362 in 2022), high poverty rates (57% of the population lives below the national poverty line), and reliance on subsistence agriculture and extractive industries (World Bank, 2023). Ranked 184th out of 193 countries on the Human Development Index (UNDP, 2023), the country grapples with significant infrastructural deficits, limited access to healthcare and education, and ongoing challenges related to post-conflict recovery following its civil war (1991–2002) as well as the 2014–2016 Ebola outbreak and the more recent COVID-19 pandemic. These factors align SL with broader sub-Saharan African (SSA) economies, which often grapple with colonial legacies, political volatility, and structural imbalances exacerbated by global commodity dependence. Like many SSA nations, SL’s labor market is characterized by a large informal sector (65% of employment), low industrialization, and youth unemployment, creating a context where organizational change initiatives must navigate resource constraints and socio-cultural complexity (AfDB, 2023).

The banking sector was selected as the focal industry for this study due to its pivotal role in Sierra Leone’s economic development and its unique position as a catalyst for organizational change. This sector comprises 14 commercial banks and has undergone significant reforms since the 2008 global crisis, including digitization (e.g., mobile banking adoption at 23% in 2023) and stricter regulatory frameworks to align with the West African Monetary Zone. Unlike agriculture and mining, which dominate Sierra Leone’s GDP, the financial services sector—though smaller—serves as a critical enabler of formal economic activity, foreign investment, and poverty reduction (AfDB, 2023). Commercial banks are central to this ecosystem, facilitating credit access for small and medium enterprises (SMEs), which employ the vast majority of the workforce, and supporting post-conflict reconstruction through infrastructure financing. By focusing on banking, the study captures a sector central to Sierra Leone’s aspirations for economic modernization, while offering insights applicable to other developing economies prioritizing financial inclusion and institutional resilience.

This study examines how transformational and transactional leadership styles interact with Sierra Leone’s cultural dimensions to influence employee performance during organizational change. Transformational and transactional leadership styles, characterized by visionary inspiration and structured incentives respectively, drive organizational change and efficiency. Their effectiveness hinges on alignment with cultural values that shape employee attitudes (Bass, 1985; Judge & Piccolo, 2004).

National business culture, a multi-layered construct that includes shared values, beliefs, and norms, exerts a deep influence on all facets of organizational life (Sertel et al., 2022). Sierra Leone’s hierarchical social structure and cultural emphasis on authority underscore the interplay of power distance and uncertainty avoidance in leadership efficacy. These dimensions shape employee expectations during organizational change, aligning cultural norms with leadership approaches that prioritize structure or adaptability (Gelfand et al., 2007; Hofstede, 2001; Kortsch et al., 2023). There has been a lot of study on the impact of national business culture on leadership, but we still do not fully grasp how these dynamics play out in emerging economies (Owusu Ansah & Louw, 2019; Ullah et al., 2022). This study seeks to contribute to this critical area of inquiry by examining the impact of transformational and transactional leadership styles on employee performance during organizational change in Sierra Leone’s commercial banking sector, with a specific focus on how national business culture moderates these relationships. By examining these complex relationships, the study will offer significant insights for practitioners, policymakers, and future studies aiming to improve leadership efficacy and drive successful organizational change in culturally diverse and dynamic environments.

## 2. Literature Review and Hypothesis

### 2.1. Leader–Member Exchange (LMX) Theory and Transformational Leadership

LMX Theory posits that leaders develop differentiated relationships with subordinates, categorizing them as “in-group” (high-quality exchanges marked by trust and mutual respect) or “out-group” (lower-quality, transactional exchanges) (Graen & Uhl-Bien, 1995). Transformational leadership (TRF) aligns closely with LMX’s in-group dynamics. TRF leaders cultivate high-quality relationships by inspiring intrinsic motivation, providing individualized support, and fostering personal growth (Bass & Riggio, 2006). These behaviors mirror LMX’s emphasis on trust and reciprocity, which enhance job satisfaction and performance (Choi, 2024). For instance, TRF components like Individualized Consideration directly reflect LMX’s focus on personalized attention, while Idealized Influence builds loyalty parallel to in-group dynamics (Avolio & Bass, 2004). Empirical studies confirm that transformational leadership positively influences organizational outcomes. It enhances employee performance by fostering a sense of purpose and drive (Bass & Avolio, 1994; Judge & Piccolo, 2004) and boosts creativity by encouraging innovation and challenging the status quo. TRF also promotes organizational citizenship behavior, as employees are more likely to go beyond formal job duties in environments built on trust and mutual respect (Podsakoff et al., 1990; Qalati et al., 2022). These outcomes highlight the transformative effect of leadership on performance by confirming that TRF-driven environments reduce out-group perceptions, promoting collective engagement and adaptive performance during organizational change (Fu et al., 2022). Given the alignment between TRF behaviors and LMX’s high-quality relational dynamics, and considering empirical evidence demonstrating TRF’s positive impact on performance and engagement during change initiatives, we hypothesize the following:

**Hypothesis 1.**  *Transformational leadership positively impacts employee performance by fostering intrinsic motivation, creativity, and adaptive behaviors during organizational change*.

#### 2.1.1. The Contingency Theory and Transactional Leadership

Contingency theory asserts that leadership effectiveness depends on situational factors such as task structure and leader–member relations (Fiedler, 1967). Task-oriented styles thrive in stable environments, while relationship-oriented styles suit ambiguous contexts. Transactional leadership (TRS) embodies the task-oriented dimension of contingency theory. TRS emphasizes structured goals, contingent rewards, and corrective interventions (Bass, 1990), making it effective in routine or high-clarity settings (e.g., regulatory compliance in banking). Studies have shown that TRS enhances task performance through extrinsic motivation and task clarity, consistent with contingency theory’s premise that structured environments benefit from directive leadership (Hamstra et al., 2014; Judge & Piccolo, 2004). Transactional leaders improve performance by establishing clear expectations, monitoring compliance, and rewarding adherence to predefined standards and this ensures stability and efficiency in routine tasks, such as meeting deadlines for regulatory reporting or minimizing errors in customer service. For instance, a supervisor offering bonuses for error-free loan processing reinforces task-focused behaviors that are critical during periods of procedural change. Some have criticized this approach for being too focused on short-term goals and for failing to inspire creativity, innovation, or long-term development in followers and can lead to limited follower engagement, as it does not stimulate personal growth or intrinsic motivation (Bass, 1990; Northouse, 2021). Despite these constraints, transactional leadership remains a significant instrument for maintaining authority and ensuring the effective execution of tasks in specific organizational contexts. Given the alignment between TRS practices and contingency theory’s emphasis on structured environments, and considering evidence that TRS drives performance through extrinsic rewards and clarity, we hypothesize the following:

**Hypothesis 2.**  *Transactional leadership fosters improvements in employee performance through extrinsic motivation, task clarity, and compliance with structured goals*.

Conversely, in unstable contexts (e.g., organizational change), TRS alone may falter, necessitating complementary TRF behaviors to inspire adaptability (Hansen & Pihl-Thingvad, 2019). Transformational leadership inspires with a compelling vision, driving long-term success and engagement (Bass & Avolio, 1994), while transactional leadership emphasizes incentives and penalties depending on performance, ensuring short-term goals and stability (Judge & Piccolo, 2004). Rather than viewing these two styles as mutually exclusive, research indicates that they can be used together to achieve organizational effectiveness (Hamstra et al., 2014). Leaders who successfully integrate both approaches can navigate diverse challenges, aligning both immediate goals and long-term visions, and optimizing the performance and well-being of their teams. Considering the complementary nature of TRF and TRS identified in prior research, and recognizing that effective leadership often requires balancing short-term stability with long-term vision, we hypothesize the following:

**Hypothesis 3.**  *There is a significant relationship between transformational and transactional leadership styles in influencing employee performance*.

#### 2.1.2. Cultural Dimensions (Hofstede) and Leadership Adaptation

Hofstede’s Cultural Dimensions Theory offers a powerful lens for exploring how cultural values shape behavior, particularly within organizational contexts. Among the six dimensions developed by Hofstede (1980b), power distance and uncertainty avoidance are adopted in this study, as they directly influence leadership styles, decision-making processes, and organizational behaviors. Understanding these dimensions is crucial for banking executives, as it allows them to tailor their management practices to align with the cultural contexts in which they operate, ultimately enhancing performance in the banking sector. The role of national culture in shaping organizational practices, management styles, and employee performance has been the subject of extensive research (Jin, 2024). A recent study by Almansoori and Ahmad (2023) discovered a substantial positive association between national culture and employee productivity, emphasizing that organizational culture, shaped by cultural values and norms, significantly influences operational procedures and outcomes. This is consistent with prior findings highlighting the role of national culture in shaping affective commitment and, ultimately, employee performance (Udin, 2019). Power distance has been shown to be key in shaping leadership effectiveness and employee outcomes. In high power distance cultures, employees expect leaders to exhibit strong influence and provide clear guidance. Transformational leadership cultivates a sense of security and aligns employees with organizational goals. Chua et al. (2023) and Gelfand et al. (2007) highlight that leaders in high power distance cultures can leverage these cultural norms to enhance alignment and performance. On the other hand, uncertainty avoidance influences how employees perceive risk and structure within the workplace. In high uncertainty avoidance cultures, employees prefer clear rules and predictable environments, making them more responsive to leadership styles that provide stability. Ma et al. (2024) found that uncertainty avoidance moderates the relationship between leadership and performance, suggesting that leaders must tailor their strategies to resonate with the cultural dynamics of their teams.

Sierra Leone’s banking sector has undergone rapid transformations, including digitization and regulatory reforms (World Bank, 2023). These changes create inherent uncertainty, making transactional leadership’s structured approach critical for stability. However, in high power distance cultures, employees may perceive transactional rewards as coercive rather than motivational during transitions, weakening its effectiveness. Therefore, recognizing power distance and uncertainty avoidance as core cultural dimensions is essential for leaders aiming to foster alignment and drive success in diverse cultural contexts. Building on existing research, the following hypotheses are put forward:

**Hypothesis 4.**  *Power distance positively moderates the relationship between transformational leadership and employee performance, such that the positive impact of transformational leadership is stronger in high power distance environments*.

**Hypothesis 5.**  *Power distance negatively moderates the relationship between transactional leadership and employee performance during organizational change, such that the positive impact of transactional leadership is weaker in high power distance environments*.

**Hypothesis 6.**  *Uncertainty avoidance negatively moderates the relationship between transformational leadership and employee performance, such that the positive impact of transformational leadership is weaker in high uncertainty avoidance settings*.

**Hypothesis 7.**  *Uncertainty avoidance positively moderates the relationship between transactional leadership and employee performance, reducing the effectiveness of transactional leadership in high uncertainty avoidance environments*.

**Figure 1:**
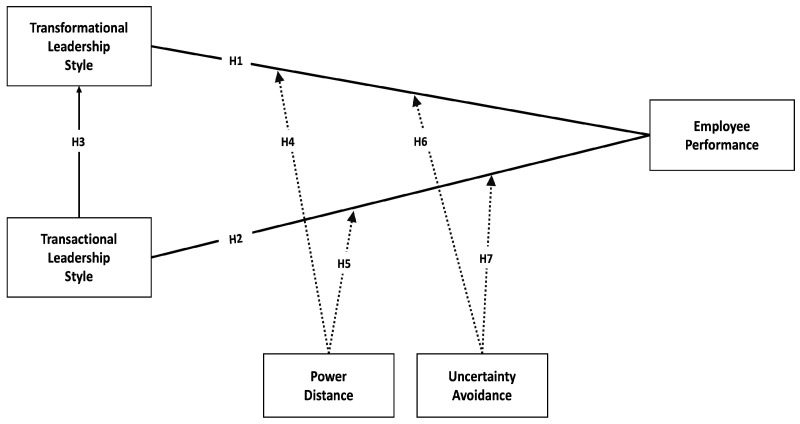
Proposed conceptual model.

Based on the above deliberation, Figure 1 shows the proposed conceptual model.

## 3. Methods

### 3.1. Study’s Participants Procedures

This study employs a cross-sectional research design to shed light on the dynamics at play between leadership behaviors, employee performance, and cultural context. The design is suitable for examining relationships because it allows data to be collected at a single point in time (Wang & Cheng, 2020). Participants were drawn from Sierra Leone’s banking sector, a critical driver of economic modernization. To ensure that participants had experienced organizational change, screening questions were included in the survey. Respondents were asked whether they had encountered significant structural, technological, or procedural changes in their organization within the past three years. This step helped to establish a baseline for change exposure, ensuring the relevance of their responses to leadership effectiveness during transitions. Questionnaires were administered in English, Sierra Leone’s official business language. A stratified random sampling technique was employed, with strata defined by bank size (large: >500 employees; medium: 200–500 employees; small: <200 employees) and department (operations, customer service, IT, finance, and HR). Participants were randomly selected from employee rosters provided by HR departments, ensuring proportional representation across strata. Of the 1200 surveys distributed, 820 valid responses were received, yielding a 68.3% response rate. Non-response was primarily due to employee turnover during data collection or incomplete surveys. Participants were assured anonymity to mitigate social desirability bias, and follow-up reminders were sent to improve participation. This sample size ensures adequate power for the analysis, especially for Structural Equation Modeling (Hair et al., 2019).

### 3.2. Measures

The measurement items were adapted to ensure cultural and contextual relevance for Sierra Leone’s banking sector. Modifications included simplifying language (e.g., ‘vision’ to ‘future goals’), aligning examples with local workplace practices (e.g., ‘bonuses’ instead of ‘rewards’), and emphasizing hierarchical norms (e.g., ‘supervisors make decisions’). Pilot testing with 30 bank employees confirmed comprehension and face validity. Adjustments preserved the original constructs’ theoretical intent while enhancing local applicability. Transformational and transactional leadership styles were assessed using ten items from the well-established Multifactor Leadership Questionnaire (MLQ) developed by Avolio and Bass (2004), a tool widely validated and used across diverse cultural contexts. Sample transformational leadership items included “My supervisor clearly explains how changes will improve our work”; sample transactional leadership items included “My supervisor gives bonuses or praise when I complete tasks correctly”’. Participants evaluated the leadership behaviors of their immediate supervisors, focusing on their experiences as subordinates. For example, frontline employees rated branch managers, while middle managers rated senior leaders. This approach ensured that responses reflected received leadership rather than self-reported leadership behavior. For the cultural dimensions (uncertainty avoidance and power distance), six items were adapted from Hofstede’s cultural dimensions framework (Hofstede, 1980b). Sample power distance items included “It is better to let supervisors make decisions without questioning them”. Sample uncertainty avoidance items included “Following strict procedures is important, even during changes’’. Employee performance was measured using five items drawn from (Koopmans et al., 2014). Sample employee performance items included “I meet my daily work targets effectively”. All responses were documented using a five-point Likert scale, varying from “strongly disagree” to “strongly agree”, thereby ensuring consistency and clarity in data collection.

### 3.3. Demographic Information

The study included 820 bank employees in Sierra Leone, with a gender split of 56% male and 44% female. Most participants (51%) were aged 26–35, held a bachelor’s degree (65%), and had 4–6 years of work experience (27%). Roles spanned frontline staff (63%), middle management (29%), and senior executives (7%), enabling an analysis of cultural dimensions across organizational hierarchies. The details can be found in Table 1.

## 4. Results

### 4.1. Introduction

For data analysis, structural equation modeling (SEM) was employed using SmartPLS software 4.1, leveraging the partial least squares (PLS) approach (Hair et al., 2019). PLS-SEM stands out for its flexibility, robustness, and ability to handle complex models, making it a preferred method for this study (Batra, 2024). The extensive utilization of this framework demonstrates its efficacy in explaining complex relationships, including the impact of leadership styles on employee performance and the moderating influence of cultural aspects. To validate the robustness of the measurement model, various studies were conducted, essential for establishing the model’s reliability and preparing for the examination of the suggested hypotheses.

### 4.2. Assessment of Measurement Model

To ensure the vigor of the measurement model, we evaluated the reliability and validity of the constructs by examining various key indicators. First, the factor loadings (FL) of each item under a construct were assessed. The widespread consensus is that factor loadings between 0.6 and 0.7 are acceptable (Hair et al., 2019). In Table 2, all factor loadings exceeded 0.7, indicating that the individual item reliability criterion was met. Next, composite reliability was checked, which is the internal consistency of a construct’s elements. According to Hair et al. (2019), a reliability threshold of 0.7 or higher is considered sufficient. Cronbach’s alpha (Ca) coefficients and composite reliability (Cr) coefficients were used to test all constructs, as presented in Table 2. The Ca coefficients ranged from 0.751 to 0.849, surpassing the 0.7 threshold and confirming the internal consistency and reliability of the measurement scales. The Cr coefficients ranged from 0.754 to 0.850, surpassing the threshold and confirming the internal consistency and reliability. Also, the Variance Inflation Factor (VIF) values for all predictors, ranged from 1.367 to 2.359, well below the common threshold of 5 (Hair et al., 2019), indicating that multicollinearity is not a concern and that the predictors are not highly correlated with one another.

For convergent validity, which ensures that items within a construct truly measure the same underlying concept, Chin (1998) recommends an Average Variance Extracted (AVE) value of 0.5 or greater. As shown in Table 2, all variables in this study exceeded the 0.5 threshold, thereby validating the convergent validity of the constructs. To evaluate discriminant validity, both the Heterotrait–Monotrait (HTMT) ratio and the Fornell–Larcker criterion were examined. The HTMT ratio, known for its robustness in testing discriminant validity, should be less than 0.9 to avoid multicollinearity or validity issues. The results, as presented in Table 3, showed HTMT ratios ranging from 0.626 to 0.773, indicating no validity concerns (Sarstedt et al., 2022). The Fornell–Larcker criterion was employed, which compares the square root of each construct’s AVE with its correlations to other constructs. As displayed in Table 3, the square root of each construct’s AVE surpassed its correlations with other constructs, further affirming discriminant validity.

### 4.3. Explanatory and Predictive Power

An important indicator of a model’s predictive power is the R^2^; it evaluates the extent to which the independent variables account for the variation in the dependent variables (Hair et al., 2019), and, as suggested by Cohen (2013), R^2^ values are typically categorized as follows: 0.25 (weak), 0.5 (moderate), and 0.75 (significant). The R^2^ for employee performance was found to be 0.777, reflecting a substantial level of explanatory power. In contrast, transformational leadership demonstrated an R^2^ of 0.520, indicating a moderate level of variance explained. These results suggest that the model performs especially well in explaining employee performance, while transformational leadership is moderately well explained by the predictors (see Table 4). In addition, Q^2^ predict evaluates the model’s predictive relevance, which shows how successful the model is at predicting future data points for the endogenous constructs (Hair et al., 2019). Further, Cohen (2013) recommends that values above 0.35 are considered indicative of substantial predictive relevance. In the present study, the Q^2^ predict value for employee performance was 0.774, demonstrating substantial predictive relevance, while transformational leadership had a Q^2^ predict value of 0.519, signifying moderate predictive relevance. These findings suggest that the model is highly capable of predicting employee performance and moderately effective in predicting transformational leadership (see Table 4).

### 4.4. Hypothesis Testing Results

**Figure 2:**
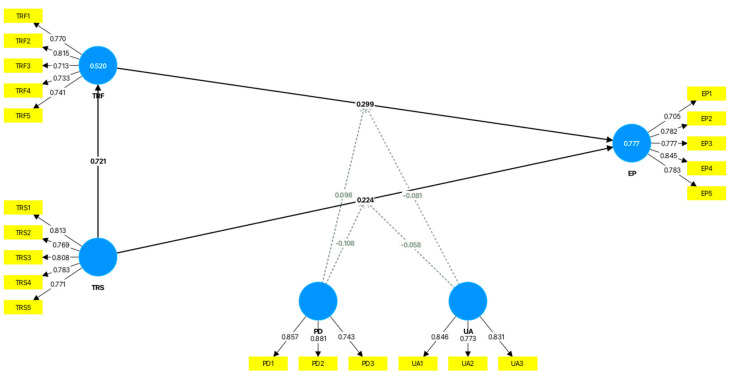
Structural model.

This study followed the approach of Hair et al. (2019) by using the bootstrapping method to determine the significance of the path coefficients. Through this method, the statistical correlations within the model were reliably examined using 5000 bootstrap samples and a robust sample size of 820. The findings, encompassing the complete estimates of the path coefficients (β), are shown in Table 5 and illustrated in Figure 2, offering an extensive overview of the structural model’s efficacy.

Hypothesis 1 tested the direct effect of transformational leadership (TRF) on employee performance (EP). The results presented in Table 5 demonstrate a significant and positive relationship between TRF and EP (β = 0.299, T = 8.998, *p* < 0.001). Hypothesis 2 examined the impact of transactional leadership (TRS) on EP. The findings indicate a significant and positive relationship between TRS and EP (β = 0.224, T = 7.557, *p* < 0.001). Hypothesis 3 explored the correlation between TRF and TRS. The result shows a strong positive correlation between these two leadership styles (β = 0.721, T = 35.515, *p* < 0.001). Hypothesis 4 assessed the moderating effect of power distance (PD) on the relationship between TRF and EP. The results show a significant and positive moderation (β = 0.098, T = 3.995, *p* < 0.001), indicating that as power distance increases, the positive effect of TRF on EP becomes stronger. Hypothesis 5 tested the moderating effect of power distance (PD) on the relationship between TRS and EP. The results demonstrate a significant and negative moderation (β = −0.108, T = 3.430, *p* = 0.001), suggesting that in high power distance environments, the positive effect of TRS on EP weakens. Hypothesis 6 evaluated the moderating effect of uncertainty avoidance (UA) on the relationship between TRF and EP. The results reveal a significant and negative moderation (β = −0.081, T = 3.520, *p* < 0.001), indicating that higher uncertainty avoidance reduces the positive impact of TRF on EP. Hypothesis 7 tested the moderating effect of uncertainty avoidance (UA) on the relationship between TRS and EP. The results show a significant and negative moderation (β = −0.058, T = 2.733, *p* = 0.006), suggesting that in high uncertainty avoidance settings, the positive effect of TRS on EP diminishes, thus rejecting H7.

### 4.5. Interaction Effects Analysis

To explore the combined effects of transformational and transactional leadership, participants were categorized into four groups (Table 6) based on median splits of TRF and TRS scores. ANOVA was conducted to compare EP across groups, with post hoc Tukey tests identifying significant differences. Cultural dimensions (power distance and uncertainty avoidance) were included as covariates to assess contextual moderators.

The findings from the 2 × 2 matrix (Table 6) revealed that significant differences emerged in EP across the four groups (F (3, 816) = 27.4, *p* < 0.001). Specifically, the post hoc tests revealed the following: High TRF/High TRS (M = 4.3, SD = 0.5) outperformed all other groups (*p* < 0.001), High TRF/Low TRS (M = 3.9, SD = 0.6) and Low TRF/High TRS (M = 3.7, SD = 0.5) did not differ significantly (*p* = 0.12), and Low TRF/Low TRS (M = 3.1, SD = 0.7) scored lowest.

The findings from the moderation analysis (Table 7) showed that High PD amplified the synergy of High TRF/High TRS (β = 0.15, *p* = 0.003), while High UA strengthened Low TRF/High TRS (β = 0.11, *p* = 0.02).

## 5. Discussion

By using the theories of Hofstede’s cultural dimensions, the contingency theory of leadership, and the Leader–Member Exchange (LMX) theory, this study seeks to further enhance our understanding of the ways in which cultural dimensions influence leadership styles in Sierra Leone.

The study demonstrates a substantial association between transformational leadership and employee performance, hence boosting motivation, job satisfaction, and organizational commitment. The results support Hypothesis 1, suggesting that commercial banks benefit from a more engaged workforce through TRF. Research consistently highlights its positive impact. Findings by Fareed et al. (2023) show that transformational leadership fosters a supportive environment that boosts performance, while García-Morales et al. (2012) emphasize its role in promoting innovation in banking. Further research by Hundie and Habtewold (2024) and C.-C. Lee et al. (2023) show that TRF thrives in dynamic environments, helping organizations to adapt in the VUCA world (Nguyen et al., 2023; Onyeneke & Abe, 2021). So, bank executives should embrace change and nurture a practice of innovation (Oroh et al., 2024).

Hypothesis 2 confirms that transactional leadership (TRS) also impacts employee performance (EP) positively, supporting studies by (Hundie & Habtewold, 2024). TRS uses incentives and penalties to meet job requirements (Aarons, 2006; Bass, 1985). However, clear organizational structures, defined performance goals, and a system of rewards and penalties are essential for improving performance within commercial banks and the positive effect TRS further stresses the idea that leadership effectiveness varies by context. Transactional leadership’s structured approach aligns with the banking sector’s need for quick, effective, and measurable outcomes (Asrar-ul-Haq & Anwar, 2018). Additionally, the 2 × 2 analysis (Table 6) further underscores the complementarity of TRF and TRS in Sierra Leone’s banking sector. Leaders who blend visionary inspiration (TRF) with structured incentives (TRS) achieve superior performance, likely because they address both intrinsic motivation (e.g., aligning with organizational goals) and extrinsic needs (e.g., clarity during change). This synergy is particularly potent in High PD cultures, where employees value authoritative guidance and clear rewards. Conversely, relying solely on one style yields suboptimal results. For instance, High TRF/Low TRS may fail to provide the stability needed in High UA contexts, while Low TRF/High TRS risks stifling innovation. These findings validate the transformational–transactional mix proposed in African leadership studies Dartey-Baah (2015) and align with Sierra Leone’s cultural duality of hierarchical respect and risk aversion.

Hypothesis 3 further elucidates the relationship between TRF and TRS, demonstrating that while these frameworks are distinct, they are not mutually exclusive. This complementary coexistence is empirically supported by Mwita et al. (2023) and Nielsen et al. (2019) who highlight their interplay in practice. Bass and Avolio (1994) argue that effective leaders blend both approaches; that is, using TRS for clear expectations and TRF to motivate employees toward higher performance. Recent research supports this synergy.

Studies by Judge and Piccolo (2004) and Klein (2023) demonstrate that integrating both leadership styles leads to better employee performance and satisfaction, while similarly, Mekonnen and Bayissa (2023) found that leaders combining both approaches enhance individual and organizational outcomes. Dartey-Baah (2015) refers to this as the “transformational-transactional mix”, particularly effective in dynamic sectors like banking, where leaders must balance routine tasks with fostering innovation. These results are consistent with the LMX theory, which emphasizes that high-quality leader–employee relationships, based on trust and respect, enhance employee performance (Erdogan & Bauer, 2015; Graen & Uhl-Bien, 1995). The combination of transactional and transformational leadership lends credence to the contingency theory of leadership, which contends that situational factors determine a leader’s effectiveness rather than a single style (Fiedler, 1967). In commercial banks, where trust and clarity are essential, this dual leadership approach proves effective. By balancing transformational motivation with transactional goal setting, leaders navigate the complexities of the financial sector, ensuring strong performance and high-quality relationships (Breevaart et al., 2014).

In Sierra Leone’s high power distance context, transformational leaders leverage respect for authority to inspire discretionary effort (e.g., employees working overtime to align with a leader’s vision). Conversely, transactional leadership’s reliance on contingent rewards may falter in such settings, as employees might perceive rewards as obligatory rather than motivational, weakening their practical efficacy (Iguisi, 2014). This contrasts with low power distance cultures, where TRS’s structured incentives may more directly enhance task performance.

Hypothesis 4 indicated that power distance positively moderates the relationship between transformational leadership and employee performance (β = 0.098, T = 3.995, *p* < 0.001, H4 supported), with higher power distance enhancing the impact of TRF. In high power distance cultures, common in many African contexts, transformational leaders can more effectively inspire and motivate employees by leveraging their authority (Ansah, 2015; De Mooij, 2019). Transformational behaviors, such as providing vision and fostering inclusivity, are especially impactful in hierarchical cultures where employees often look to leaders for direction (M. C. C. Lee & Ding, 2023). A strong leader can significantly influence team dynamics in high power distance settings (Peng et al., 2020), and transformational leaders are likely to build a more committed workforce, critical for performance in banking (Jiatong et al., 2022). These results emphasize the prominence of cultural hierarchy in shaping leadership effectiveness, suggesting that Sierra Leone’s banks can benefit from leadership practices tailored to their cultural context.

Conversely, power distance is found to negatively moderate the relationship between transactional leadership and employee performance (β = −0.108, T = 3.430, *p* = 0.001, H5 supported), weakening the positive effects of TRS as power distance increases. As Gelfand et al. (2007) note, the effectiveness of transactional leadership depends on the cultural context. In high power distance cultures, such as those in many African countries, transactional practices often fail to motivate employees, who may view rewards and recognition as mere compliance rather than genuine incentives for better performance (Iguisi, 2014).

Additionally, Tetteh et al. (2023) found that cultural dimensions, especially power distance, significantly moderate the relationship between non-monetary motivation factors and employee performance and this creates a disconnect between leaders and employees, reducing TRS’s effectiveness. In high power distance cultures, employees may feel less empowered to take initiative or voice concerns, leading to lower engagement and diminished responses to transactional rewards and punishments.

Hypothesis 6 corroborates that uncertainty avoidance negatively moderates the relationship between transformational leadership and employee performance, supporting Hypothesis H6. In high uncertainty avoidance cultures, employees fancy structured environments and clear guidelines, which conflict with the change-oriented nature of transformational leadership. Further, Den Hartog and De Hoogh (2024) found that resistance to change can emerge over time, undermining the effectiveness of transformational leadership and negatively impacting performance outcomes. Similarly, the GLOBE project by House et al. (2004) highlighted that high uncertainty avoidance cultures hinder TRF, as employees in these environments are less comfortable with ambiguity and dynamism. In Sierra Leone, where uncertainty avoidance is a prominent cultural factor, employees may feel uneasy with the risk-taking and ambiguity of TRF, leading to lower motivation and performance.

Finally, we unexpectedly found that uncertainty avoidance positively moderates the relationship between transactional leadership (TRS) and EP (β = −0.058, T = 2.733, *p* = 0.006), leading to the rejection of Hypothesis 7. Previous research suggests that employees with high uncertainty avoidance typically respond well to transactional leadership, which provides clear directions and reduces ambiguity (DeChurch, 2019). An overemphasis on transactional leadership styles may stifle creativity and development in the long run (Dong, 2023). These findings highlight the complexity of leadership dynamics across cultural contexts, suggesting that high uncertainty avoidance may not always lead to the expected outcomes in the TRS context. Nonetheless, the study highlights the relevance of Hofstede’s cultural dimensions theory in relation to power distance and uncertainty avoidance. High power distance cultures, like Sierra Leone, influence the effectiveness of transformational leadership, with leaders leveraging their authority to inspire and motivate employees more effectively. Similarly, the uncertainty avoidance dimension explains how employees in such cultures may resist change, making transformational leadership less effective in environments where clear structures and risk aversion are more valued (Hofstede, 1980b). These cultural dimensions further underscore the importance of tailoring leadership styles to the cultural context, supporting Hofstede’s argument that cultural values shape leadership effectiveness. Therefore, understanding these cultural factors is crucial for leaders in navigating the complexities of high power distance and high uncertainty avoidance cultures to enhance employee performance.

## 6. Conclusions

The findings from this study corroborate those from previous research showing that TRF and TRS have a beneficial effect on EP. Previous studies confirm that TRF enhances motivation and organizational commitment, while TRS improves performance through clear structures and rewards, especially during change (Hundie & Habtewold, 2024; Jun & Lee, 2023; C.-C. Lee et al., 2023). This study extends the literature by highlighting the moderating role of cultural dimensions (power distance and uncertainty avoidance) in leadership effectiveness. These findings support the work of Hofstede (1980a) and Gelfand et al. (2007), demonstrating that high power distance strengthens transformational leadership, while high uncertainty avoidance makes transactional leadership more effective due to employees’ preference for structure. This research expands the literature by underscoring the importance of tailoring leadership styles to cultural values, particularly in high power distance and high uncertainty avoidance settings like Sierra Leone. By combining these approaches, leaders can better navigate these cultural complexities, enhancing employee performance and organizational success in dynamic environments like the banking sector.

### 6.1. Contributions of the Study

#### 6.1.1. Theoretical Contributions

There is a substantial theoretical advance in this paper by examining leadership styles through the lens of cultural dimensions, with a focus on power distance and uncertainty avoidance. Traditional leadership models, such as transformational and transactional leadership, often assume their universal applicability across cultures (Dickson et al., 2003). This study challenges that view by showing that leadership effectiveness is shaped by cultural factors that influence how leadership behaviors are perceived and their impact on employee performance (Dong, 2023). By incorporating PD and UA as moderators, the study advocates for a culturally adaptive approach to leadership. In High PD settings like Sierra Leone, transformational leadership proves more effective as employees, who are more accustomed to hierarchical authority and respond better to visionary leadership.

Conversely, in High UA environments, transactional leadership thrives by providing the structure and predictability that employees value in uncertain contexts. This contests the notion of universal leadership models, highlighting the necessity of including cultural values and norms in the application of leadership theories (Crede et al., 2019; Jung & Avolio, 1999). The study also refines the transformational–transactional leadership model by demonstrating how these styles complement each other in culturally diverse, hierarchical, and High UA settings. Instead of viewing these leadership styles as distinct, the research suggests they can be strategically blended, with leaders shifting between the two to inspire change (transformational) and ensure compliance (transactional) (Dartey-Baah, 2015). This corresponds with contingency theory, which asserts that leadership efficacy is contingent upon contextual factors.

The findings hold relevance for sub-Saharan African contexts sharing SL’s socio-economic profile, such as high power distance, post-colonial institutional legacies, and reliance on transactional banking models. For instance, Ghana and Nigeria similarly exhibit hierarchical organizational cultures and banking sectors undergoing digitization amid low financial inclusion (AfDB, 2023). By demonstrating how cultural dimensions moderate leadership efficacy in SL, this study provides a framework for leaders in comparable SSA economies to tailor strategies that harmonize global best practices with local norms.

Finally, there is a substantial theoretical advance in this paper by examining leadership styles through the lens of cultural dimensions, with a focus on power distance and uncertainty avoidance. It calls for global leadership frameworks that incorporate cultural dimensions to address the diverse realities of organizations worldwide.

#### 6.1.2. Practical Contributions

This research offers significant practical insights, especially in the context of leadership development and organizational effectiveness in culturally diverse environments. It highlights that leaders should be sensitive to the cultural traits of their employees and adapt their leadership approaches accordingly, especially in high power distance and high uncertainty avoidance settings. In such environments, leadership development programs can integrate strategies that train leaders to leverage transformational leadership to inspire commitment while utilizing transactional leadership to provide clarity, stability, and predictability. With this two-pronged approach, leaders may attend to both immediate operational needs and the organization’s long-term objectives, aligning with employees’ cultural expectations. In High PD environments, leaders can use their authority to foster trust and present a compelling vision for change, reducing resistance by aligning employee behavior with the directives of respected leaders. In contrast, in High UA environments, transactional leadership helps to alleviate employee anxiety by providing clear guidelines, structured incentives, and stability, all of which employees in these contexts highly value. This ensures smoother transitions during organizational change and enhances overall employee engagement and productivity.

The research further highlights that transformational leadership in High PD contexts can build trust and inspire alignment with organizational goals, while transactional leadership in High UA settings provides the structure necessary for success in uncertain environments. This culturally aligned leadership approach not only meets both emotional and operational needs but also reduces resistance, increasing employee buy-in during times of change. For multinational organizations, these findings offer a framework for developing cross-cultural leadership strategies. In High PD countries, emphasizing transformational leadership can cultivate respect and alignment with organizational values, while in High UA cultures, structured transactional approaches can help to ensure consistency and reduce ambiguity. Additionally, HR policies can be informed by these insights, encouraging culturally sensitive recruitment, training, and performance management practices. Companies can design policies that assess cultural adaptability in recruitment, recognize leaders who demonstrate cultural competence, and reward practices that improve leadership effectiveness across global and diverse contexts.

Leaders should adopt a dual-style approach use transformational behaviors (e.g., articulating change visions) to inspire commitment and adaptability and complement it with transactional practices (e.g., milestone-based rewards) to ensure task adherence and reduce uncertainty. Training programs should emphasize contextual flexibility—for example, prioritizing TRF-TRS integration in High PD branches (e.g., rural areas) while leaning on TRS in High UA departments (e.g., compliance teams). By adapting leadership styles to fit cultural expectations, organizations can foster a more motivated, engaged, and productive workforce, driving greater success in both local and international markets.

### 6.2. Limitations of the Study

Several limitations are included in this investigation. First, the study’s exclusive focus on Sierra Leone’s banking sector limits the generalizability of findings to other industries or regions with distinct cultural and operational contexts. Additionally, the reliance on self-reported measures introduces potential biases, such as social desirability and recall bias, which may affect the accuracy of responses. While the study prioritized power distance and uncertainty avoidance due to their relevance to Sierra Leone’s hierarchical and risk-averse culture, other dimensions of Hofstede’s framework (e.g., individualism/collectivism, masculinity/femininity) were not examined, potentially overlooking additional cultural influences. Furthermore, although the study confirmed employees’ exposure to organizational change, it did not differentiate between types of change (e.g., technological vs. structural). While the 2 × 2 analysis offers novel insights, median splits may oversimplify leadership style continua. Future studies should employ latent profile analysis or longitudinal designs to capture dynamic style interactions and should explore how specific change contexts interact with leadership styles. The lack of causal inferences inherent to cross-sectional data underscores the need for longitudinal studies to assess the long-term effects of culturally adaptive leadership strategies.

### 6.3. Suggestions for Future Research

Subsequent research ought to investigate the significance of these findings across different industries and regions, such as healthcare or education, to broaden insights on culturally adaptive leadership. Comparative studies across countries or regions could identify universal and culturally specific leadership principles. Additionally, investigating the interplay of several cultural factors and the significance of cultural intelligence in leadership efficacy will provide significant new insights. Longitudinal studies could track the long-term effects of culturally aligned leadership on employee performance. A cross-country study in the West African region, such as comparing Sierra Leone with other countries within the sub-Saharan Africa region, could further contextualize these findings and offer region-specific strategies for leadership and change.

## Figures and Tables

**Table 1:** Demographic information.

Demographic	Category	Frequency (n)	Percentage (%)
Gender	Male	459	56%
	Female	361	44%
Age	18–25 years	86	10%
	26–35 years	415	51%
	36–45 years	251	31%
	46–55 years	57	7%
	56+ years	11	1%
Education	Certificate	7	1%
	Diploma	91	11%
	BSc	529	65%
	Masters	190	23%
	PhD	3	0%
Work Experience	Below 1 year	78	10%
	1–3 years	208	25%
	4–6 years	222	27%
	7–10 years	145	18%
	10+ years	167	20%
Job Function	Frontline (e.g., tellers, customer service)	520	63%
	Middle Management (e.g., branch managers)	240	29%
	Senior Management (e.g., executives)	60	7%
Hierarchical Level	Entry-level	510	62%
	Supervisor	210	26%
	Manager	90	11%
	Total	820	100%

**Table 2:** Reliability and validity.

Construct	Factor Loading (FL)	Cronbach’s Alpha (Ca)	Composite Reliability (Cr)	Average Variance Extracted (AVE)	Variance Inflation Factor (VIF)
Transformational Leadership		0.813	0.832	0.570	
TRF1	0.77				1.979
TRF2	0.815				1.755
TRF3	0.713				1.500
TRF4	0.733				1.596
TRF5	0.741				1.865
Transactional Leadership		0.849	0.850	0.623	
TRS1	0.813				1.908
TRS2	0.769				1.773
TRS3	0.808				1.999
TRS4	0.783				1.705
TRS5	0.771				1.688
Power Distance		0.773	0.807	0.687	
PD1	0.857				1.919
PD2	0.881				1.800
PD3	0.743				1.376
Uncertainty Avoidance		0.751	0.754	0.668	
UA1	0.846				1.646
UA2	0.773				1.367
UA3	0.831				1.611
Employee Performance		0.839	0.844	0.608	
EP1	0.705				1.394
EP2	0.782				1.966
EP3	0.777				1.974
EP4	0.845				2.359
EP5	0.783				1.621

Source: developed by the authors.

**Table 3:** Discriminant validity.

HTMT Ratio	EP	PD	TRF	TRS	UA
EP					
PD	0.822				
TRF	0.850	0.810			
TRS	0.842	0.729	0.857		
UA	0.765	0.614	0.639	0.587	
**Fornell and Larcker’s Criterion**	**EP**	**PD**	**TRF**	**TRS**	**UA**
EP	0.780				
PD	0.683	0.829			
TRF	0.745	0.652	0.755		
TRS	0.731	0.601	0.721	0.789	
UA	0.613	0.480	0.517	0.474	0.817

Source: developed by the authors.

**Table 4:** Explanatory and predictive power of constructs (EP and TRF).

Construct	R-Square	Q^2^ Predict
EP	0.777	0.774
TRF	0.520	0.519

Source: developed by the authors.

**Table 5:** Hypothesis testing.

Relationships	Path Coefficient (β)	T Statistics	Probability (*p*) Values
H1: TRF → EP	0.299	8.998	0.000
H2: TRS →EP	0.224	7.557	0.000
H3: TRS →TRF	0.721	35.515	0.000
H4: PD × TRF → EP	0.098	3.995	0.000
H5: PD × TRS → EP	−0.108	3.430	0.001
H6: UA × TRF → EP	−0.081	3.520	0.000
H7: UA × TRS → EP	−0.058	2.733	0.006

Source: developed by the authors.

**Table 6:** The 2 × 2 leadership style interaction analysis.

Leadership Style Group	N	Mean EP	SD	Comparison	Mean Difference	*p*-Value
High TRF/High TRS	230	4.3	0.5	vs. High TRF/Low TRS	0.4	<0.001
High TRF/Low TRS	180	3.9	0.6	vs. Low TRF/High TRS	0.2	0.12
Low TRF/High TRS	210	3.7	0.5	vs. Low TRF/Low TRS	0.6	<0.001
Low TRF/Low TRS	200	3.1	0.7	-	-	-
ANOVA Results		F (3, 816) = 27.4	*p* < 0.001	post hoc (Tukey HSD)	-	-

Source: developed by the authors.

**Table 7:** Moderation effects (cultural dimensions).

Moderator	Interaction	Beta (β)	T-Stat	*p*-Value	Interpretation
Power Distance (PD)	PD × High TRF/High TRS → EP	0.15	3.01	0.003	High PD strengthens TRF-TRS synergy.
Uncertainty Avoidance (UA)	UA × Low TRF/High TRS → EP	0.11	2.45	0.02	High UA enhances TRS effectiveness alone.

Source: developed by the authors.

## Data Availability

The data used to support the findings of this study are available from the corresponding author upon request.
